# Therapeutic synergy of Triptolide and MDM2 inhibitor against acute myeloid leukemia through modulation of p53-dependent and -independent pathways

**DOI:** 10.1186/s40164-022-00276-z

**Published:** 2022-04-16

**Authors:** Qinwei Chen, Suqi Deng, Manman Deng, Yuanfei Shi, Mengya Zhong, Lihong Ding, Yuelong Jiang, Yong Zhou, Bing Z. Carter, Bing Xu

**Affiliations:** 1 Department of Hematology, The First Affiliated Hospital of Xiamen University and Institute of Hematology, School of Medicine, Xiamen University, Xiamen, 361102 People’s Republic of China; 2 Key Laboratory of Xiamen for Diagnosis and Treatment of Hematological Malignancy, Xiamen, 361102 China; 3 Department of Hematology, Nanfang Hospital, Southern Medical University, Guangzhou, 510515 Guangdong China; 4 Institute of Hematology, School of Medicine, Jinan University, Guangzhou, 510632 China; 5 Department of Pathology, The First Affiliated Hospital of Xiamen University and College of Medicine, Xiamen University, Xiamen, 361102 China; 6 Section of Molecular Hematology and Therapy, Department of Leukemia, The University of Texas MD Anderson Cancer Center, Houston, TX USA

**Keywords:** Acute myeloid leukemia (AML), p53, MDM2 inhibitor, Triptolide, MYC, ATF4, ER stress

## Abstract

Dysregulation of MDM2, a p53 negative regulator, frequently occurs in acute myeloid leukemia (AML) and is associated with unfavorable prognoses, rendering the p53-MDM2 axis an attractive target for the development of small-molecule inhibitors. MDM2 antagonists have been intensely developed but only lead to limited clinical activity, suggesting combination with additional drugs is an unmet medical need. In this study, we reported that Triptolide synergized with MDM2 inhibitor Nutlin-3a to suppress cell proliferation and induce mitochondrial-mediated apoptosis in p53 wt AML in vitro and ex vivo. More importantly, Triptolide cooperated with Nutlin-3a to delay tumor growth and abrogate leukemia burden in an AML xenograft model*.* In addition, we observed that Triptolide and Nutlin-3a were also cooperative in part of p53 deficient cases. Mechanistically, Nutlin-3a upregulated the transcriptional expressions of the p53 downstream targets PUMA and p21, while Triptolide declined the mRNA levels of two anti-apoptotic factors, XIAP and Mcl-1, in p53 wt cells. These effects were more notable when Triptolide and Nutlin-3a were combined. Our results revealed that Triptolide monotherapy exerted its antileukemia effect via both p53-dependent and independent ways, with the latter through perturbation of the MYC-ATF4 axis-mediated ER stress. Collectively, these data suggested that the Triptolide-Nutlin-3a combination might be a novel potential therapeutic intervention for patients with AML and it warrants further clinical evaluations.

**Supplementary Information:**

The online version contains supplementary material available at 10.1186/s40164-022-00276-z.

To the Editor,

Despite recent advances in the treatment of acute myeloid leukemia (AML), a subset of patients remains incurable, suggesting an unmet effective therapeutic need [1]. Dysfunction of p53, a renowned tumor suppressor, is often associated with poor outcomes in AML, thus activation of p53 roles is intensely studied [2, 3]. Many studies endeavor to pharmacological inhibition of MDM2, a p53 negative regulator, while p53 activation with MDM2 inhibitor alone only results in limited clinical activities in AML [4, 5]. Thus, how to potentiate the anti-AML efficacy of MDM2 inhibitor is worthy of further investigation.

Triptolide, a traditional Chinese medicine, exerts potent anti-tumor activities in many malignant disorders [6]. Others and our prior studies have reported that low-dose (LD) of Triptolide could sensitize AML to multiple conventional chemotherapies and targeted agents [7–9]. In this study, we sought to examine the antileukemia effects of LD Triptolide in conjunction with MDM2 inhibitor Nutlin-3a on AML. Our results showed that LD Triptolide (~ 20 nM) synergized with Nutlin-3a to inhibit cell proliferation, promote caspase-cascades apoptosis, and increase nuclear instability in wild-type p53 (p53 wt) cells (Fig. 1A, B; Additional file 1: Fig. S1A–D). The synergy was evidenced by the combination index (CI) < 1, ranging from 0.18 to 0.89. Mitochondrial membrane potential (MMP) usually collapses before intrinsic apoptosis takes place. We found that LD Triptolide cooperated with Nutlin-3a to induce an obviously decreased MMP levels, indicating that mitochondrial-mediated apoptosis partially contributes to the synergistic effect (Additional file 1: Fig. S2A, B). In agreement with these in vitro data, LD Triptolide cooperated with Nutlin-3a to induce apoptosis in primary p53 wt AML cells, and to slow tumor growth and ameliorate leukemia burden in mice without unacceptable adverse effects (Fig. 1C–F; Additional file 1: Fig. S2C, D; Additional file 2: Table S1). Mechanically, Nutlin-3a transcriptionally upregulated p53 downstream targets (p21 and PUMA) rather than p53 (Fig. 1G). LD Triptolide downregulated MDM2 mRNA levels and decreased the transcriptional abundances of two anti-apoptotic components, Mcl-1 and XIAP (Fig. 1H).

**Fig. 1:**
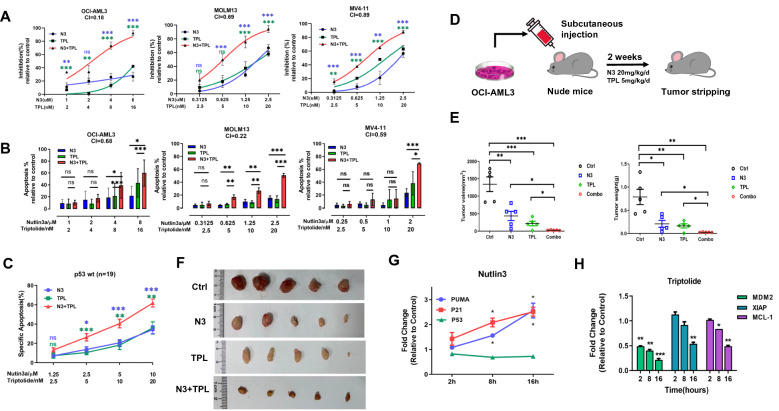
The synergistic interaction of LD Triptolide and MDM2 inhibitor in p53 wt AML cells. AML cell lines harboring wild type p53 were treated with control, Nutlin-3a (N3), LD Triptolide (TPL) or their combination for 48 h (OCI-AML3, MV4-11) or 24 h (MOLM13). **A** Cell viability was determined with the CCK-8 kit. **B** Annexin-V/PI dual staining was used to analyze cell apoptosis. A schematic plan for the in vivo study. **C** Analysis of apoptotic cells in p53 wt primary AML patients (n = 19). **D** A schematic plan for the in vivo study. **E** Tumor volumes and weight of each group were separately measured. **F** Images of subcutaneous tumors were captured at the end of the experiment. **G**, **H** qRT-PCR analysis of the mRNA levels of p53, PUMA, P21, MDM2, XIAP and MCL-1 in OCI-AML3 cells exposed to Nutlin-3a or LD Triptolide treatment for 2, 8 and 16 h. Data were presented as mean ± S.D. ns indicates not significant, *p < 0.05, **p < 0.01, ***p < 0.001, colored by corresponding single drug treatment in statistical tests

To ascertain the role of p53 status in this combined regimen, we silenced p53 using shRNA in p53 wt cells and found that Nutlin-3a failed to induce apoptosis in the p53 knockdown cells. Knocking down p53 only partially impeded the proapoptotic activity of LD Triptolide monotherapy or in combination with Nutlin-3a (Fig. 2A), implying that p53-independent molecular bases might play roles in Triptolide cytotoxicity. We indeed confirmed this implication, as Triptolide exhibited a robust cytotoxicity against all five p53 deficient primary AML cells and three p53 deficient cell lines (Fig. 2B, C; Additional file 2: Table S1). The synergistic effect of Triptolide and Nutlin-3a was observed in Kasumi-1 carrying p53 p.R248Q mutation and 2 out of 5 p53 deficient primary AML cells, while the synergy was not evident in p53 null cells(THP-1 and HL-60) (Fig. 2B, C; Additional file 2: Table S1).

**Fig. 2:**
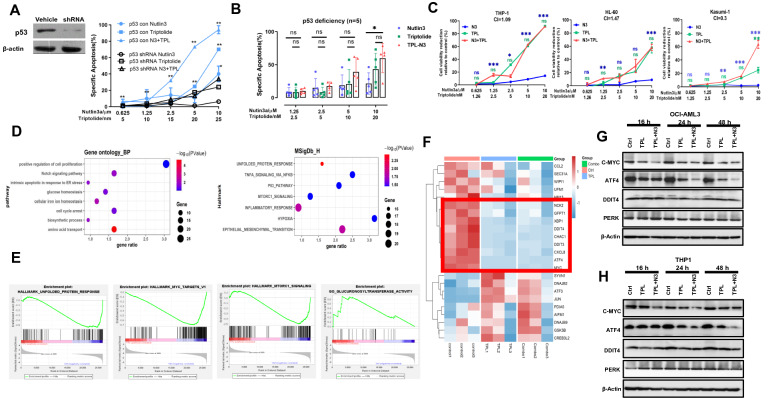
The p53 independent role of Triptolide in p53 deficent AML cells. **A** Apoptotic Analysis of OCI-AML3 cells with vehicle (red lines) or p53 shRNA (blue lines) treated with control, Nutlin-3a, Triptolide, or their combination for 24 h. **B** Analysis of apoptotic cells in p53 deficient primary AML patients (n = 5) treated as indicated doses for 48 h. **C** Cells viability was detected in AML p53 mut/null cell lines treated as indicated doses for 48 h (mut: Kasumi-1; null: THP-1, HL-60). **D** The GO and hallmark pathway analysis was performed to reveal the enriched signaling for the 482 DEGs. **E** GSEA analysis of the annotations related to ER stress pathway. **F** Heatmap displayed hierarchical clustering of ER related genes among control,Triptolide alone or Triptolide in combination with Nutlin-3a. **G**, **H** Immune blot assessed the protein levels of MYC and ATF4 in OCI-AML3 (p53 wt) and THP-1 (p53 deficiency) cells exposed to LD Triptolide and Nutlin-3a alone or in combination. ns indicates not significant, *p < 0.05, **p < 0.01, ***p < 0.001, colored by corresponding single drug treatment in statistical tests

To better understand the p53 independent function involved in Triptolide treatment, we performed RNA-Seq in OCI-AML3 cells treated with Triptolide with or without Nutlin-3a. We identified 482 shared differential expression genes (DEGs, fold change > 2, FDR < 0.05) (Additional file 1: Fig. S3A, B; Additional file 3) and observed that Triptolide significantly attenuated the activity of several pathways related to protein synthesis processes (UPR, mTORC1, MYC targets) (Fig. 2D, E). The ER stress associated genes, especially MYC and ATF4 targeted subsets (e.g., MYC, ATF4, TRIB3, CHAC1, DITT4), were remarkably blocked by Triptolide and the combined treatment (Fig. 2F). The proto-oncogene MYC enhances protein synthesis that is essential to induce an adaptive ER stress response(unfolded protein response, UPR) in tumor progression [10]. As a vital piece of UPR, PERK-eIF2a-ATF4 signaling is selectively activated to clear misfolded proteins with MYC hyperactivation [10, 11]. Our subsequent functional assays demonstrated that MYC and ATF4 was extremely suppressed by Triptolide and its combination with Nutlin-3a, while PERK and DDIT4 exhibited no significant changes in both OCI-AML3 and THP-1 cells. Interestingly, the synergistic effect was observed in MYC deletion at early stage(16 hs) and in ATF4 deletion at later stage(24–48 h) of treatment (Fig. 2G, H). We suppose that ATF4 probably served as a key UPR effector in response to Triptolide treatment.

In summary, our study offered a novel potent therapeutic regimen by combining LD Triptolide with MDM2 inhibitor against p53 wt AML. More importantly, this drug combination was also active in some AML models with p53 deficiencies. The synergism was closely related to activation of p53 relevant proapoptotic cascades in p53 wt AML. Beyond the p53 reliant functions, Triptolide also exerted its antileukemia efficacy through interfering with the MYC-ATF4 axis, a p53 independent pathway. These data provided a potential rationale for clinical administration for the treatment of patients with AML [12], while prospective studies are warranted to further validate this hypothesis.

## Supplementary Information

**Additional file 1: Fig. S1** The synergistic interaction of LD Triptolide and MDM2 inhibitor in p53 wt AML cells. **(A)** The representative flow plots of cell apoptosis. **(B)** Fluorescence analysis of DAPI staining of OCI-AML3 cells treated with TPL and Nutlin-3a alone or in combination. **(C)** Immunoblotting examination of caspase 3 and PARP in OCI-AML3 and MV4-11 cell lines treated with Nutlin-3a (10 uM for AML3, 2uM for MV4-11), TPL (20 nM) alone or in combination for 24 h. **(D)** Analysis of Annexin-V + cells of OCI-AML3 cells treated as indicated with or without a v-ZAD-FMK pretreatment. **(E)** Analysis of apoptotic cells in health donors (n = 4). **Fig. S2**. The synergistic interaction of LD Triptolide and MDM2 inhibitor in p53 wt AML cells. **(A)** The representative flow plots of JC-1 fluorescence signal. Red fluorescence: aggregates; green fluorescence: means monomers. **(B)** Bar plot shows the reduction of MMP (MFI (red channel)/ MFI (green channel) *100%) after 48 h drug treatment. **(C)** Body weight analysis of AML xenografts treated with different treatment groups. **(D)** Tumors sections were prepared and stained by H.E. for histological examination. Data were presented as mean ± S.D. ns indicates not significant, * *p* < 0.05, ** *p* < 0.01, *** *p* < 0.001. **Fig. S3.** The p53 independent role of Triptolide. OCI-AML3 cells were treated with LD Triptolide (TPL) or the combination therapy of LD Triptolide plus Nutlin-3a for 24 h, then were referred to perform the RNA-seq. **(A)** Volcano graphs showed the significant upregulated genes (red) and downregulated genes (blue) in the TPL (left panel) or the combined group (right panel). **(B)** Venn graph indicated the overlapping 482 DEG genes.

**Additional file 2: Table S1.** Clinical Characteristics of primary patients.

**Additional file 3. **Additional materials and methods.

## Data Availability

The datasets and materials analyzed during the current study are available from the corresponding author upon reasonable request.
