# Supplementary figures and images for: Therapeutic synergy of Triptolide and MDM2 inhibitor against acute myeloid leukemia through modulation of p53-dependent and -independent pathways
Source: Exp Hematol Oncol. 2022 Apr 16;11:23. doi: 10.1186/s40164-022-00276-z (PMC9013083; doi:10.1186/s40164-022-00276-z)

**Fig. S1**

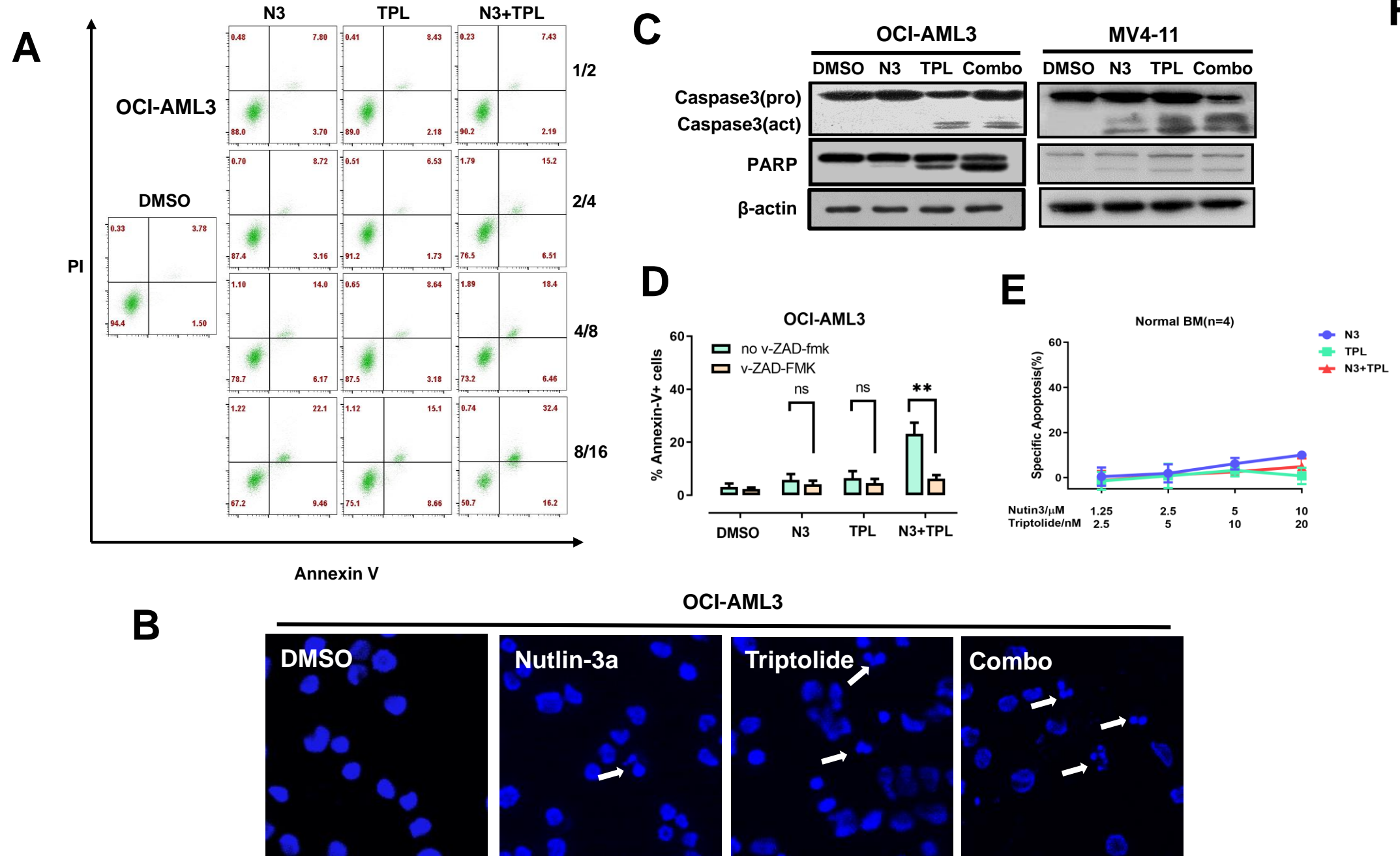

Fig. S2

A

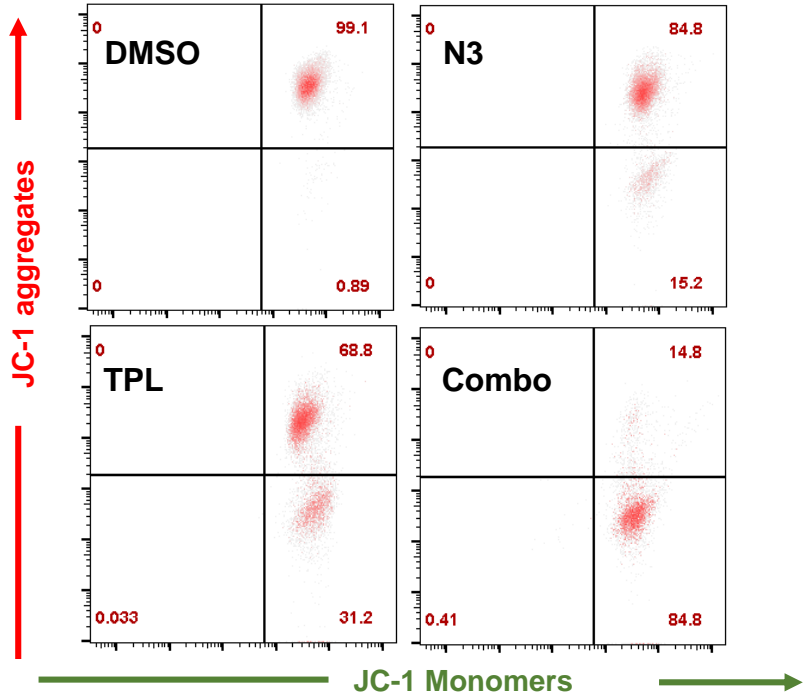

B

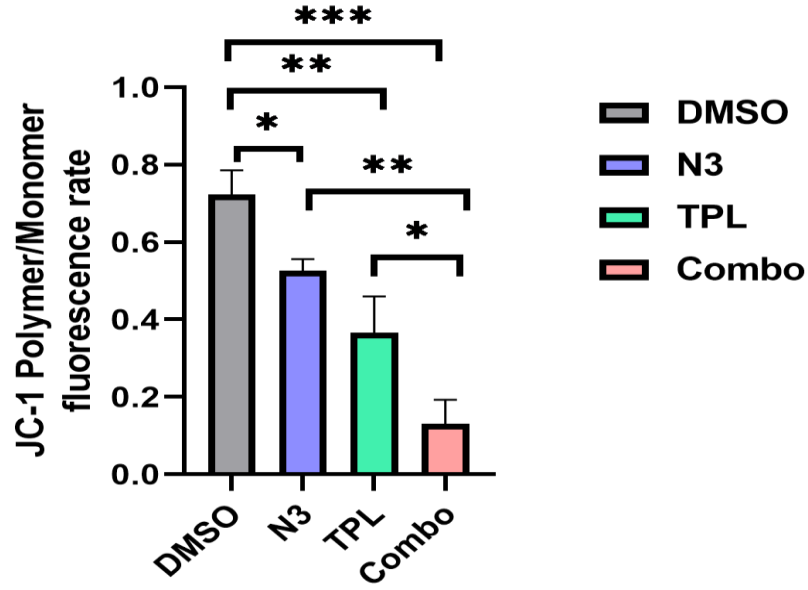

C

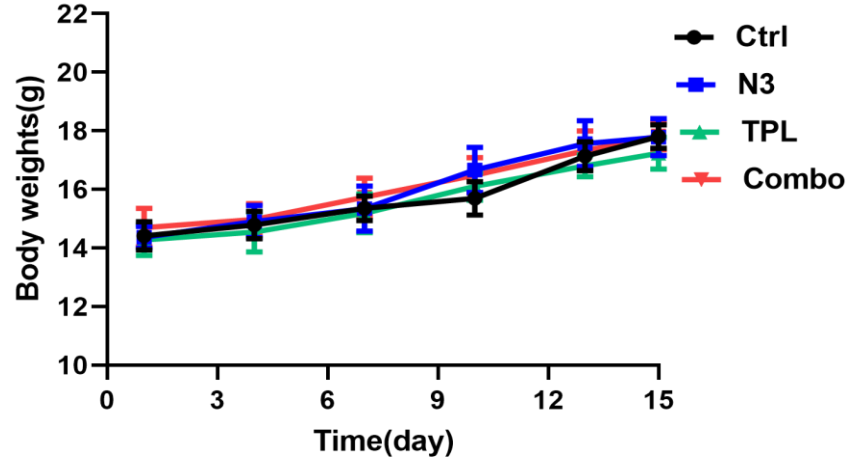

D

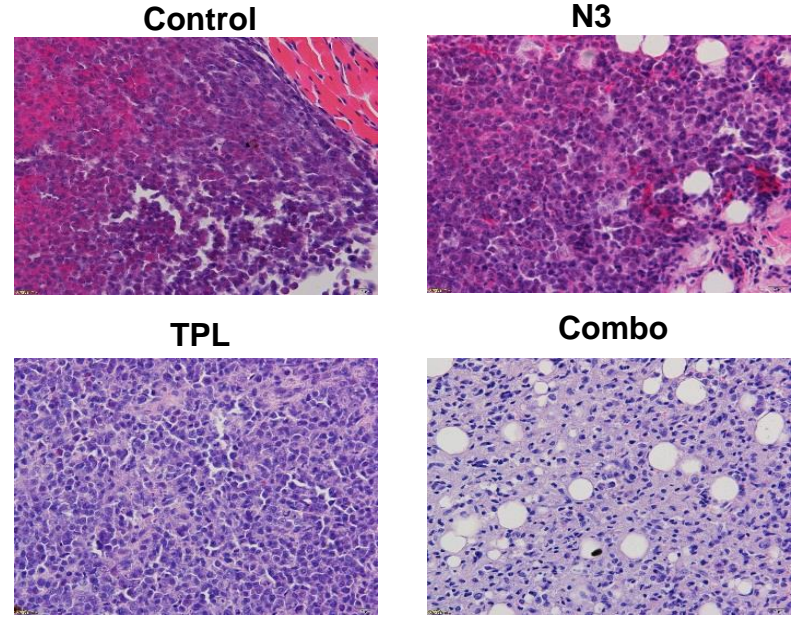

Fig. S3

A

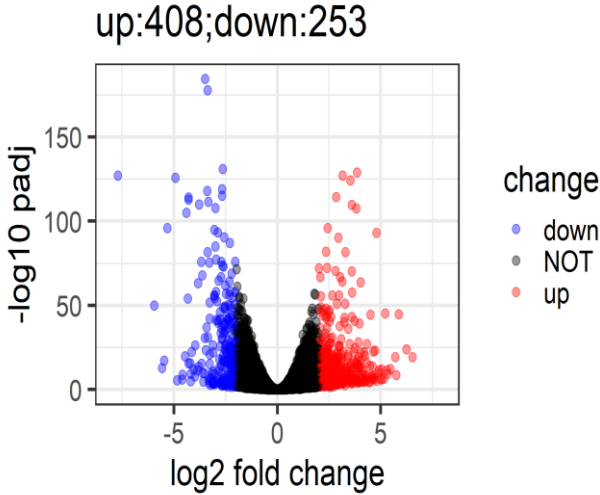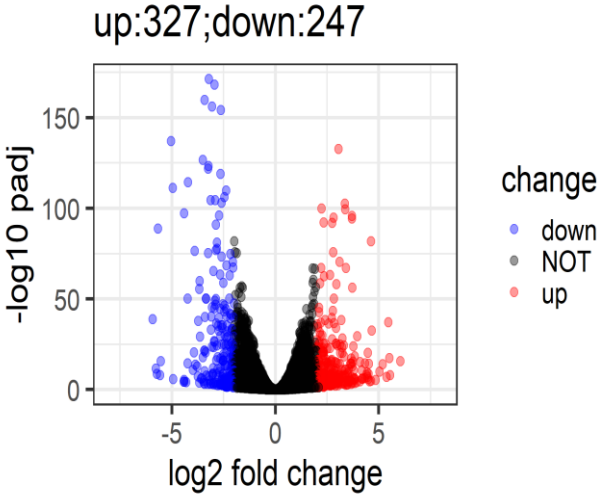

B

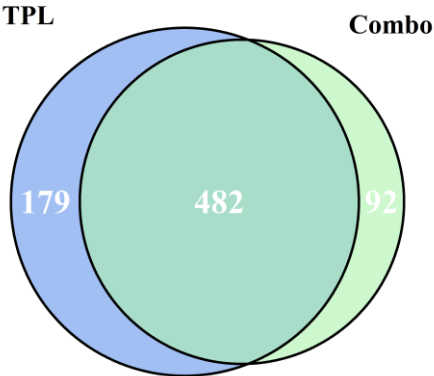

Supplement: Supplementary file 1 — Additional file 1: Fig. S1 The synergistic interaction of LD Triptolide and MDM2 inhibitor in p53 wt AML cells. (A) The representative flow plots of cell apoptosis. (B) Fluorescence analysis of DAPI staining of OCI-AML3 cells treated with TPL and Nutlin-3a alone or in combination. (C) Immunoblotting examination of caspase 3 and PARP in OCI-AML3 and MV4-11 cell lines treated with Nutlin-3a (10 uM for AML3, 2uM for MV4-11), TPL (20 nM) alone or in combination for 24 h. (D) Analysis of Annexin-V + cells of OCI-AML3 cells treated as indicated with or without a v-ZAD-FMK pretreatment. (E) Analysis of apoptotic cells in health donors (n = 4). Fig. S2. The synergistic interaction of LD Triptolide and MDM2 inhibitor in p53 wt AML cells. (A) The representative flow plots of JC-1 fluorescence signal. Red fluorescence: aggregates; green fluorescence: means monomers. (B) Bar plot shows the reduction of MMP (MFI (red channel)/ MFI (green channel) *100%) after 48 h drug treatment. (C) Body weight analysis of AML xenografts treated with different treatment groups. (D) Tumors sections were prepared and stained by H.E. for histological examination. Data were presented as mean ± S.D. ns indicates not significant, * p < 0.05, ** p < 0.01, *** p < 0.001. Fig. S3. The p53 independent role of Triptolide. OCI-AML3 cells were treated with LD Triptolide (TPL) or the combination therapy of LD Triptolide plus Nutlin-3a for 24 h, then were referred to perform the RNA-seq. (A) Volcano graphs showed the significant upregulated genes (red) and downregulated genes (blue) in the TPL (left panel) or the combined group (right panel). (B) Venn graph indicated the overlapping 482 DEG genes. [file 40164_2022_276_MOESM1_ESM.pdf]
