# Supplementary material for: Therapeutic synergy of Triptolide and MDM2 inhibitor against acute myeloid leukemia through modulation of p53-dependent and -independent pathways
Source: Exp Hematol Oncol. 2022 Apr 16;11:23. doi: 10.1186/s40164-022-00276-z (PMC9013083; doi:10.1186/s40164-022-00276-z)
Supplement: Supplementary file 2 — Additional file 2: Table S1. Clinical Characteristics of primary patients. [file 40164_2022_276_MOESM2_ESM.docx]

**Table S1. Clinical Characteristics of primary patients.**

|  | | | | | | | molecular mutations | | | |  |  |  |
| --- | --- | --- | --- | --- | --- | --- | --- | --- | --- | --- | --- | --- | --- |
| No. | Sex | Age | FAB | WBC | Blast (%) | Karyotype | *NPM1* | *FLT3-ITD* | *P53 status* | others | induction therapy | status after induction therapy | CI |
| 1 | F | 46 | AML-M2 | 8.1 | 52 | 46, XX | － | － | *Wt* | － | IA | NR | <1 |
| 2 | F | 40 | AML-M2 | 275.3 | 92 | 46, XX | + | － | *Wt* | *DNMT3A* | IA | NR | <1 |
| 3 | F | 29 | AML-M2 | 154.73 | 74 | 46, XX | － | － | *Wt* | *WT1* | IA | NR | <1 |
| 4 | M | 26 | AML M5b | 3.15 | 96 | 46, XY, del(11)(q23) | － | － | *Wt* | *CEBPA、WT1、KMT3a* | CLAG | NR | <1 |
| 5 | F | 47 | AML-M3 | 7.1 | 43 | 46, XX | － | － | *Wt* | *CEBPA* | IA | CR | <1 |
| 6 | M | 32 | AML-M4 | 10.66 | 30.5 | 46, XY | － | － | *Wt* | *c-Kit* | IA | CR | >1 |
| 7 | M | 40 | AML-M2 | 29.52 | 68.5 | 46, XY | － | － | *Wt* | *CEBPA* | IA | CR | >1 |
| 8 | F | 16 | AML-M2 | 18.2 | 24 | 46, XX | － | － | *Wt* | *－* | IA | NR | >1 |
| 9 | M | 54 | AML-M1 | 76.76 | 86 | 46,XY | ＋ | － | *Wt* | *CEBPA* | IA | PR | >1 |
| 10 | M | 52 | AML-M2 | 29.43 | 61 | 46, XX | － | － | *Wt* | *AML1-ETO、Kit* | IA | CR | <1 |
| 11 | M | 34 | AML-Mix | 8.66 | 75 | 46, XY | － | － | *Wt* | *CEBPA* | IA+VP | NR | <1 |
| 12 | F | 17 | AML-M5 | 55.4 | 46.5 | 46,XX，del(11)(q23) | － | － | *Wt* | *MLL/AF6* | IA | NR | <1 |
| 13 | M | 39 | AML-M2 | 17.75 | 64.9 | 45,X,-Y,t(8;21)(q22;q22)[7]/46,XY[3] | － | － | *Wt* | *AML1-ETO* | IA | － | >1 |
| 14 | M | 24 | AML-unclassified | 99.4 | 21 | 46, XY | － | － | *Wt* | *－* | DA | NR | <1 |
| 15 | F | 33 | AML-M1 | 27.37 | 94.5 | 46, XX | － | － | *Wt* | *－* | IA | NR | <1 |
| 16 | M | 58 | AML from MDS | 94.81 | 62.5 | 46, XY | － | － | *Wt* | *DNMT3A、RUNX1* | CAG | NR | <1 |
| 17 | M | 47 | AML-M5 | 29.83 | 76.2 | 46,XX | － | － | *Wt* | DNMT3A、IDH1 | IA | NR | <1 |
| 18 | F | 64 | AML-M5 | 3.64 | 12.5 | 47,XY,+21[20] | － | － | Wt | － | AZA+Bcl2i | － | >1 |
| 19 | F | 66 | AML-M2 | 106.61 | 20.5 | 46,XY | － | － | Wt | WT1 | AZA+Bcl2i | CR | <1 |
| 20 | M | 41 | AML-M5b | 14.55 | 75 | 46, XY，t(11;15)(q23;q12) | － | － | *Null* | *MLL* | IA | NR | <1 |
| 21 | F | 55 | AML-M2 | 8.66 | 37.5 | 46，XX，del(5)(q14q33)[4]/47`49,idem,add(2)(p13),+8,+11,+14,-17,+mar[cp16] | － | － | *Mt* | *EGR1、MLL* | IA | NR | <1 |
| 22 | F | 54 | AML-M2 | 71.9 | 64 | 46, XX | － | － | *Null* | *－* | DA | NR | >1 |
| 23 | F | 34 | AML-M2 | 0.65 | 40 | 46,XY | － | － | *Mt* | *－* | AZA+HAG | CR | >1 |
| 24 | F | 85 | AML-M2 | 22.56 | 20.2 | 44,XY,del(5)(q13q31),der(12)(?),-13,der(14)t(13:14)(q21;p12),-16[10] /43,idem,der(11)t(5;11)(q22;q25),-17[10] | － | － | *Mt* | TEL-ABL1、WT1 | IA | CR | >1 |
