# Supplementary material for: Therapeutic synergy of Triptolide and MDM2 inhibitor against acute myeloid leukemia through modulation of p53-dependent and -independent pathways
Source: Exp Hematol Oncol. 2022 Apr 16;11:23. doi: 10.1186/s40164-022-00276-z (PMC9013083; doi:10.1186/s40164-022-00276-z)
Supplement: Supplementary file 3 — Additional file 3. Additional materials and methods. [file 40164_2022_276_MOESM3_ESM.docx]

**Materials and Methods**

**Cell lines and Reagents**

Human AML cell lines (OCI-AML3, Molm-13, MV4-11, Kasumi-1, THP-1, and HL60) were purchased from ATCC (Rockefeller, sinozhongyuan, MD, USA) and cultured in RPMI-1640 medium (HyClone, Thermo Scientific, Waltham, MA, USA) supplemented with 10% fetal bovine serum (FBS, HyClone, Thermo Scientific) and 100 U/mL penicillin and 100 mg/mL streptomycin at 37℃ in 5% CO^2^ incubator. OCI-AML3, Molm-13 and MV4-11 are p53 wild type cells; Kasumi-1 is p53 mutated cells; THP-1 and HL-60 are p53 null cells.

Both Triptolide (Cat. No. S3604) and Nutlin-3a (Cat. No. S1061) were purchased from Selleck Chemicals (Houston, TX, USA). The two reagents were dissolved in dimethyl sulfoxide (DMSO; Invitrogen, Carlsbad, CA, USA) as 10 mM stock solution and stored at -20℃, which was then diluted to the designated concentrations with RPMI-1640 medium. Pan-caspase inhibitor Z-VAD-fmk was purchased from MCE (Cat.No. HY-16658).

**Primary Samples**

Bone marrow samples from AML patients (n=24) and peripheral blood specimen from healthy donors (n = 4) were obtained from the NanFang hospital, Southern Medical University, China. This study was conducted in accordance with the Declaration of Helsinki and approved by the Ethics Review Board of the Nanfang Hospital. Clinical characteristics of patients with AML are summarized in Additional file 2. Mononuclear cells were isolated with density gradient centrifugation using Lymphoprep TM (BD, Franklin Lakes, NJ, USA) and were cultured in Iscove's Modified Dulbecco's medium (HyClone, Thermo Scientific, Waltham, MA, USA) supplemented with 1 × Penicillin/ streptomycin and 10% FBS for limited time.

**Cell viability assay**

Cell Counting Kit-8 (CCK-8, Cat.NO HY-K0301, MCE, USA) was used to determine cell viability of all tested AML cells exposed to various treatment regimens. In brief, AML cells (2×10^4^/well) were seeded in 100μl medium on 96-well plates (Corning, Cat.NO CLS3595) and treated with vehicle or designated concentrations of drugs for 24 or 48h. CCK-8 reagent (10μl/well) was then added and incubated for additional 2 hours at 37℃ in 5% CO_2_ incubator. The absorbance at 450 nm was detected by a microplate reader (ELx800, BioTek, Winooski, VT, USA). Cell proliferation inhibitory rate was measured with the following formula: inhibition rate (%) = [1 − (absorbance of experimental group − absorbance of blank well)/ (absorbance of control group- absorbance of blank well)] × 100%. The data were obtained from three independent triplicates and presented as mean ± SD. Statistical analysis and IC_50_ determination were calculated by GraphPad 8.3.0.

The combination index (CI) was calculated using CompuSyn 1.0 (ComboSyn Inc., Paramus, NJ, USA) according to the Chou-Talalay method, with definition for additive effect (CI = 1), synergism (CI < 1), and antagonism (CI > 1).

**Analysis of apoptosis**

Cells were cultured and exposed to LD Triptolide with or without Nutlin-3a for 24 or 48h as described above. Cell apoptosis was assessed with Annexin V- FITC and PI (Cat.NO BMS500FI-100, eBioscience, Thermo Scientific) double staining for 15 minutes at in 4℃ in dark following the manufacturer’s instructions. Cells were then analyzed through flow cytometry (FACS Fortessa, BD Biosciences, Franklin lakes, NJ, USA). Annexin V positive cells were defined as apoptotic cells.

**Immunofluorescent staining**

Immunofluorescence with DAPI staining (4083S, Cell Signaling Technology, USA) was performed to detect nuclear status of AML cells after 48h treatment with Triptolide and Nutlin-3a alone or in combination following the manufacturer's instructions. Slides were photographed by confocal microscope (FluoView-FV1000, Olympus, Japan).

**Mitochondrial membrane potential (MMP) assay.**

OCI-AML3 cells (2×10^5^/ml) were cultured in 24-well plates and treated with Triptolide (20nM) and Nutlin-3a (10μM) alone or in combination for 24h. Cells were harvested and stained with JC-1 kit (Byeotime, Shanghai, China) for 30 minutes at 37℃. After washed with medium, mitochondrial membrane potential (MMP) was assayed with flow cytometry (FACS Fortessa, BD Biosciences, Franklin lakes, NJ, USA).

**Western blot analysis**

Whole cell extracts were subjected to Western blot analysis using primary antibodies and secondary HRP-conjugated antibodies (1:10,000, Abcam, Cambridge, UK). The primary antibodies included those against caspase-3（9662S, CST), PARP (9532S, CST), c-MYC(18583S, CST), ATF4(ET1612-37, HUABIO), DDIT4(ER1706-76, HUABIO), PERK(ER64553, HUABIO). The primary antibodies were diluted with 5% BSA -TBST. β-actin (1:1000, 4970s, CST, USA) was used as a loading control. Proteins were then visualized using an ECL Western Blotting Detection Kit (GeneFlow, Staffordshire, UK).

**Real-time** **polymerase chain reaction**

After total RNA was extracted and mRNA purified, mRNA was reverse transcripted to cDNA using the TransScripr First-Strand cDNA Synthesis SuperMix (TransScript, CA.#AT301, Beijing, China). The assays-on-demand primers and probes and TaqMan Universal Master Mix were used to examine gene expression using the Roche LC480 Sequence Detection System (TransStart) according to the user’s manual. Primer sequence are：

Mcl-1 For AGACCTTACGACGGGTTGG, Rev AATCCTGCCCCAGTTTGTTA

MDM2 For TCTAGGAGATTTGTTTGGCGT, Rev TCACAGATGTACCTGAGTCC

XIAP For CCCAAATTGCAGATTTATCAACG, Rev TGCATGTGTCTCAGATGGCC

p53 For CTTTGAGGTGCGTGTTTGTG, Rev GTGGTTTCTTCTTTGGCTGG

Puma For ATGCCTGCCTCACCTTCATC, Rev TCACACGTCGCTCTCTCTAAACC

p21 For AGCGATGGAACTTCGACTTTG, Rev CGAAGTCACCCTCCAGTGGT

β-actin For TGTGGCATCCACGAAACTAC, Rev GGAGCAATGATCTTGATCTTCA

β-actin was used as housekeeping control.

**RNA sequencing**

Cells were treated with Triptolide, Nutlin-3a, or combination for 24h. Sequencing libraries were made from poly-A RNA, and sequenced using illumine Hiseq 2500. RNA-seq paired-end reads were assessed for quality using FastQC (v0.11.6), trimmed as appropriate using Trim Galore (v0.4.1), then aligned to the human genome (Gencode GRCh38.p13, hg38) using the Hisat2(v2.1.0). Total aligned reads per gene were counted using featureCounts (v1.6.0). DESeq2 (v3.11) was performed for differential expression analysis (significantly changing expression was defined as an FDR-corrected p-value ≤ 0.005). Raw counts were normalized to TPM (Transcripts Per Kilobase of exon model per Million mapped reads) values for comparing the mRNA expression.

**AML Xenograft in Mice**

The animal study was approved by the Laboratory Animal Ethics and Management Committee of Xiamen University. A total of 20 Balb/c nude mice (4-5 weeks; female; Beijing, HFK bioscience Co. Ltd.) were subcutaneously implanted with 9 x 10^6^ of OCI-AML3 cells, respectively. After three days, AML engrafted mice were randomized into four groups (5 mice per group): vehicle (PBS), Triptolide (0.5 mg/kg/d), Nutlin-3a (20 mg/kg/d), and Triptolide plus Nutlin-3a group. Body weight was monitored daily. AML xenografted mice were euthanized upon tumor size reaching ~1500 mm^3^, and the subcutaneous tumors from the four groups were then photographed. At the end of the experiment, tumor weight of each mouse was measured and recorded. Tumor volume was calculated using the formula: V=1/2(L*W^2) (L: length, W: width). Histological examination was performed on tumor sections with H.E staining.

**Statistical analysis**

Values represent the mean ± SEM for at least three independent experiments. All statistical analyses were performed using GraphPad Prism 8.3.0 software. Variables between two groups were compared using the t test(equal variance) or Welch’s t test. Comparisons among multiple groups were performed using the One-way analysis of variance (ANOVA) followed by the Bonferroni post hoc test. P < 0.05 was considered as statistically significant.
